# Exploring variation of coverage and access to dental care for adults in 11 European countries: a vignette approach

**DOI:** 10.1186/s12903-022-02095-4

**Published:** 2022-03-09

**Authors:** Juliane Winkelmann, Jesús Gómez Rossi, Falk Schwendicke, Antoniya Dimova, Elka Atanasova, Triin Habicht, Kaija Kasekamp, Coralie Gandré, Zeynep Or, Úna McAuliffe, Liubove Murauskiene, Madelon Kroneman, Judith de Jong, Iwona Kowalska-Bobko, Katarzyna Badora-Musiał, Sylwia Motyl, Gonçalo Figueiredo Augusto, Peter Pažitný, Daniela Kandilaki, Lubica Löffler, Carl Lundgren, Nils Janlöv, Ewout van Ginneken, Dimitra Panteli

**Affiliations:** 1 Department of Healthcare Management, Technische Universität Berlin, H 80, Straße des 17. Juni 135, 10623 Berlin, Germany; 2 Charité Universitätsmedizin, Department of Oral Diagnostics, Digital Health and Health Services Research, Aßmannshauser Straße 4-6, 14197 Berlin, Germany; 3 Medical University – Varna, 55 Marin Drinov str, Varna, 9002 Bulgaria; 4 WHO Barcelona Office for Health Systems Financing, Sant Pau Art Nouveau Site (La Mercè pavilion), Sant Antoni Maria Claret, 167, 08025 Barcelona, Spain; 5 University of Tartu, Ülikooli 18, 5090 Tartu, Estonia; 6 Institute for Research and Information in Health Economics (IRDES), 117, bis Rue Manin, 75019 Paris, France; 7 Oral Health Services Research Centre and School of Public Health, University College Cork, Cork, T12K8AF Ireland; 8 Department of Public Health, Institute of Health Sciences, Faculty of Medicine, Vilnius University, M. K. Čiurlionio g. 21/ 27, 03101 Vilnius, Lithuania; 9 Nivel, Netherlands Institute for Health Services Research, Otterstraat 118, 3513 CR Utrecht, The Netherlands; 10 Faculty of Health Science, Institute of Public Health, Jagiellonian University Medical College, ul. Skawińska 8, 31-066 Kraków, Poland; 11 Institute of Dentistry, Faculty of Medicine, Jagiellonian University Medical College, Kraków, Poland; 12 Public Health Research Centre, National School of Public Health, Nova University Lisbon, Rua da Junqueira, 100, 1349-008 Lisbon, Portugal; 13 Prague University of Economics and Business, W. Churchill Sq. 1938/4, 130 67 Prague 3, Žižkov, Czech Republic; 14 Independent Health Care Expert, Bratislava, Slovakia; 15 Vardanalys, Drottninggatan 89, 113 60 Stockholm, Sweden; 16 European Observatory on Health Systems and Policies, WHO European Centre for Health Policy, Eurostation (Office 07C020), Place Victor Horta/Victor Hortaplein, 40/10, 1060 Brussels, Belgium

**Keywords:** Dental care, Coverage, Access, Cost-sharing, Benefits, Financial protection

## Abstract

**Background:**

Oral health, coupled with rising awareness on the impact that limited dental care coverage has on oral health and general health and well-being, has received increased attention over the past few years. The purpose of the study was to compare the statutory coverage and access to dental care for adult services in 11 European countries using a vignette approach.

**Methods:**

We used three patient vignettes to highlight the differences of the dimensions of coverage and access to dental care (coverage, cost-sharing and accessibility). The three vignettes describe typical care pathways for patients with the most common oral health conditions (caries, periodontal disease, edentulism). The vignettes were completed by health services researchers knowledgeable on dental care, dentists, or teams consisting of a health systems expert working together with dental specialists.

**Results:**

Completed vignettes were received from 11 countries: Bulgaria, Estonia, France, Germany, Republic of Ireland (Ireland), Lithuania, the Netherlands, Poland, Portugal, Slovakia and Sweden. While emergency dental care, tooth extraction and restorative care for acute pain due to carious lesions are covered in most responding countries, root canal treatment, periodontal care and prosthetic restoration often require cost-sharing or are entirely excluded from the benefit basket. Regular dental visits are also limited to one visit per year in many countries. Beyond financial barriers due to out-of-pocket payments, patients may experience very different physical barriers to accessing dental care. The limited availability of contracted dentists (especially in rural areas) and the unequal distribution and lack of specialised dentists are major access barriers to public dental care.

**Conclusions:**

According to the results, statutory coverage of dental care varies across European countries, while access barriers are largely similar. Many dental services require substantial cost-sharing in most countries, leading to high out-of-pocket spending. Socioeconomic status is thus a main determinant for access to dental care, but other factors such as geography, age and comorbidities can also inhibit access and affect outcomes. Moreover, coverage in most oral health systems is targeted at treatment and less at preventative oral health care.

**Supplementary Information:**

The online version contains supplementary material available at 10.1186/s12903-022-02095-4.

## Introduction

Oral diseases, such as dental caries (tooth decay), periodontal disease (gum disease) and edentulism (tooth loss) are persistently among the most prevalent conditions globally, despite being largely preventable [1]. They can have significant consequences, including unremitting pain, sepsis, reduced quality of life, lost school days, disruption to family life, and decreased work productivity. As such, they pose a substantial health and economic burden for individuals, families and society as a whole [2, 3]. Routine access to primary oral health care allows for early detection and management of oral diseases and can mitigate their negative impacts [4]. The importance of oral health has received increased attention over the past few years. Both the 74th World Health Assembly Resolution (2021) and The Lancet Issue on Oral Health (2019) have highlighted the need to shift away from the traditional curative approach towards prevention, while also integrating oral health within primary health care systems and universal health coverage programmes [2–5].

Despite the significant impact of oral health on general health and well-being, many countries restrict dental benefits covered by the statutory health system to specific treatments or age groups [6, 7]. Several dental care services require either cost-sharing or are paid fully out-of-pocket. There are large disparities in levels of cost-sharing and types of treatments excluded from the benefit basket across national and even regional jurisdictions. At the same time, there is increasing evidence that limited coverage reduces both financial protection and people’s capacity to obtain dental care if they cannot pay for treatment or disposables [8, 9]. This leads to inequalities in access to dental health services within and across countries and eventually to inequities in oral health [5, 7, 10, 11]. A 2019 survey on areas of care where access might be a problem in European countries identified oral health as one area with major gaps in coverage and access [12].

Modifications to the benefit basket and how related services are financed and delivered will inevitably be needed in most countries to achieve better coverage and integration of dental care. For such efforts to be successful, it is equally important to identify and understand barriers to accessing dental care services beyond coverage, such as the physical availability and accessibility of the necessary care providers or potential differential experiences due to social determinants of health. The variation in coverage and other access barriers to dental care services across countries, however, remains under-investigated [7, 8, 12]. Studies focusing on the coverage of dental care for older adults in high-income countries [7, 12] have shown that while most countries include some coverage for oral health services in their benefit baskets, important barriers to access exist. To the best of our knowledge, a comprehensive attempt to describe dental care coverage and capture potential access barriers for the general adult population in European countries using a qualitative approach was lacking.

Against this backdrop, the aim of this paper is to compare differences in dental care coverage and access for adults in 11 European countries using a vignette approach. The three most frequent oral diseases (dental caries, periodontal disease and edentulism [3]) were chosen as the basis for the vignettes. Together, they amounted to approximately 0.75% of total disability-adjusted life years (DALYs) and 2.2% of years lived with disability (YLDs) globally in 2019 [14]. On the basis of patient pathways for each of these three conditions, we first examine which dental care services are covered under the statutory benefit package, under which conditions and to what extent (i.e. scale of user charges in the form of cost-sharing or private payments) across those countries. We then compare further barriers to realised access, such as the physical availability of dental care services.

This research was carried out as part of the work for the Expert Group on Health System Performance Assessment (HSPA) of the European Commission, aiming to explore the usefulness of the patient vignette approach as a complementary tool for identifying gaps and challenges in access to health care in the context of HSPA [15].

## Methods

### Conceptual framework

A vignette is a short description of a person or situation designed to simulate key features of a real-world scenario [16–19]. A vignette case generally specifies a hypothetical patient’s age, gender, medical complaint, and health history. As a research tool, vignettes are usually presented to relevant professionals to solicit their hypothetical response or behaviour. In medical literature, vignettes are mostly used to study variations in decision-making processes, including clinical judgments made by health professionals [20, 21]. Recently, vignettes have, for example, also been used to investigate the availability and nature of certain types of care such as outpatient mental care [22] and community dementia care [23]. This study focuses on gaps in access during an episode of care that can be compared across countries. Therefore, the vignettes also include a delineation of the recommended care pathway and a list of services that could then be used to benchmark and compare access across countries.

To compare coverage and access to the services included in each vignette, we use the framework of the Gaps in Coverage and Access survey [13, 24], which explores the three traditional dimensions of coverage (population coverage, service coverage (which benefits are covered) and cost coverage (what proportion of costs is covered)) as well as a fourth dimension, labelled service access. Population coverage was not listed separately for this work, as gaps in statutory health coverage would be picked up under the service coverage dimension. In terms of service access, gaps could result from (i) a lack of physical availability of services, due to long distances to the provider, lack of sufficient statutory/contracted providers, poor quality of services, limited opening hours, waiting times and waiting lists; (ii) a person’s inability to obtain necessary care, due to their incapacity to formulate a care request, obtain the care or to apply for coverage (and fulfil the necessary requirements) because of their condition or situation (e.g. people with cognitive impairment, mentally ill, homeless), and (lack of) ability to navigate the system (such as being referred from one provider to another); and (iii) the attitude of the provider due to discrimination (on age, gender, race, religious beliefs, sexual orientation, etc.), for instance, leading to denial of care or the inability to accommodate care to the patient’s preferences [13]. Furthermore, a list of determinants that could improve or worsen access, including patient characteristics (e.g. age, sex, and socioeconomic status, insurance status, legal status, place of residence) as well as other factors (night vs. day treatment protocols), were added to the conceptual framework and respondents were also asked to provide any other determinants they thought could affect access for the vignette.

### Participant selection

Experts in 11 countries, including Bulgaria, Estonia, France, Germany, Republic of Ireland (Ireland), Lithuania, Netherlands, Poland, Portugal, Slovakia and Sweden were invited to participate in the vignette survey. The countries were selected to capture a variation of health systems (i.e., social health insurance vs. tax-financed, multi- vs. single payer, centralised vs. decentralised) and ensure geographical distribution. Depending on the country, vignettes were completed either by health services researchers knowledgeable on dental care, dentists, or by teams consisting of a health systems expert working together with dental specialists.

### Data collection: design of dental care vignettes and survey

The vignettes were designed in collaboration with the Department of Oral Diagnostics, Digital Health and Health Services Research at the Charité Medical University in Berlin (Germany). Each vignette and the corresponding care pathway represent a common realistic dental problem, with potential treatment options based as much as possible on common practice and international guidelines or recommendations. To shape each vignette, recommendations found in systematic reviews or developed by national, European or international organisations in the field of dentistry were used.

Three dental care vignettes were designed that illustrate typical care pathways for adult patients with the most common oral health conditions (caries, periodontal disease, edentulism).

*Vignette 1* explores coverage and potential access barriers in the treatment of dental caries that can be addressed by both non-restorative and restorative treatment using different materials (e.g. non-restorative: regular application of fluoride, gels, varnishes or sealants, or a combination thereof, resin infiltration; restorative: fillings using dental amalgams or composite resins, crowns) [25–28].

*Vignette 2* focuses on periodontal conditions caused by plaque induced inflammation of the gingivae and characterised by red swollen tissues and bleeding (gingivitis) with periodontitis, resulting in further loss of supporting bone and attachment. Recommended treatment includes patient instruction on daily plaque removal as well as the removal of supra-gingival plaque, calculus, stain (dental cleaning) and sub-gingival deposits (root planning) and control of local plaque retentive factors [29, 30]. The removal of dental calculus, which is part of the scaling and root planning treatment, also presents a very effective (primary and secondary) preventive intervention for periodontal disease.

*Vignette 3* considers coverage and access challenges for edentulous patients. Edentulous patients have a choice among different rehabilitation options: while complete dentures are widely used, the use of implant-borne replacements is increasing and there is evidence supporting their benefit in minimizing bone resorption. Prosthetic dental work is costly, but different modalities may be more or less affordable to patients [31, 32].

Table 1 presents the three vignettes, including relevant services. Each vignette describes the patient, their symptoms and potential care decisions for their clinical situation. The sequence of services corresponds to the usual care pathway, which might not necessarily be the same for all countries and settings. It was expected that the chosen services might not reflect standard practice in some participating countries, and respondents were invited to describe these differences.

**Table 1:** Dental care vignettes—patient description and services in patient pathway

Vignettes	Services
*Vignette 1: Urgent care with root canal and prosthodontic treatment* A 35-year-old patient has not been able to sleep for two nights due to a strong, beating pain in the right-lower jaw. The patient requests an urgent dental appointment. The dentist determines that the patient needs a root-canal treatment to preserve the first lower molar, and treat the pain. The patient decides for the root canal treatment and against the alternative of tooth extraction. Following the root canal treatment, reconstruction with composite (filling) material is used until a fixed prosthodontic treatment (crown/onlay) can be placed	Emergency consultation with dentist
	Radiography ((bitewing) X-rays)
	Root canal treatment **OR** Tooth extraction
	(interim) reconstruction with white filling material
	Fixed prosthodontic treatment (crown/onlay)
*Vignette 2: Periodontal treatment* A 66-year-old patient with co-morbidities (obesity, diabetes) has frequent discomfort in the upper jaw. After a consultation, chronic periodontitis with generalized level 2 mobility is diagnosed, requiring scaling and root planning, involving periodontal probing and elimination of dental calculus and frequent follow-up visits to stop disease progression and stabilize bone-loss	Scheduled visit with the dentist
	Scaling and root planning (performed by a dentist)
	Periodontal probing, and elimination of dental calculus (performed by dental assistant or hygienist)
	Regular follow-up visits
*Vignette 3: Implant-borne restoration and prosthetic rehabilitation* An edentulous 75-year-old patient received full upper and lower dentures 5 years ago. She feels she has lost significant capacity to chew as the lower prosthesis is poorly retained and gets displaced when speaking or eating. She seeks counseling from her dentist, who recommends two implants on the lower anterior jaw and an overdenture to improve retention. She agrees with this course of treatment and against more sophisticated fixed alternatives	Consultation and surgical planning
	Surgical implantation
	Prosthetic rehabilitation: New prosthesis or adjustment of old prosthesis using the implants **OR** (Partially) fixed dentures

To collect the data, a survey was constructed which presented each vignette in a separate table outlining all individual services per vignette (Table 1). In addition, for each service, experts were asked to indicate statutory service coverage (which benefits are covered) and cost coverage (what proportion of costs is covered). Moreover, for the access dimension, they were asked to indicate physical availability of services, a person’s ability to obtain care, providers’ attitude and any additional determinants they thought could affect access for each service of the vignettes. The full survey tool is available online (Additional file 1: Table S4).

#### Data analysis and reporting

**Fig. 1:**
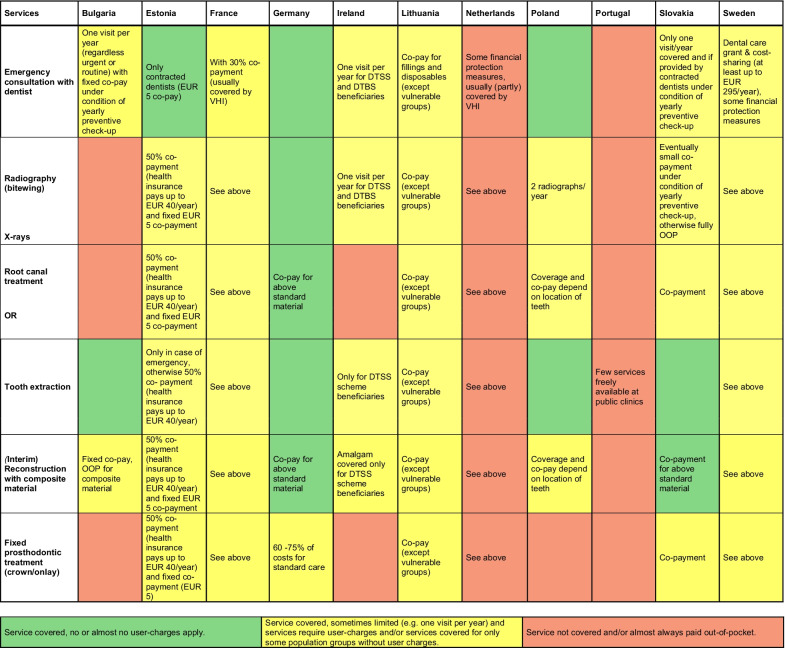
Coverage across countries for services provided in Vignette 1: Root canal and prosthodontic treatment (35-year-old patient). *Notes:* empty cells summarise information using the traffic light system; *DTSS* Dental Treatment Services Scheme, *DTBS* Dental Treatment Benefit Scheme, *OOP* out-of-pocket payments; *VHI* voluntary health insurance

**Fig. 2:**
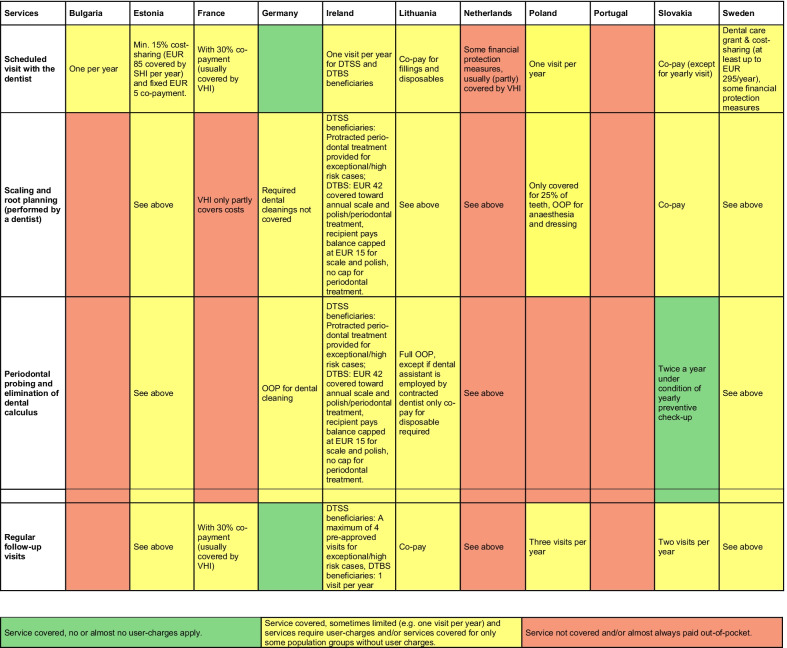
Coverage across countries for services provided in Vignette 2: Periodontal treatment (66-year-old patient). *Notes:* empty cells summarise information using the traffic light system; *DTSS* Dental Treatment Services Scheme; *DTBS* Dental Treatment Benefit Scheme; *SHI* Social health insurance; *OOP* out-of-pocket payments; *VHI* voluntary health insurance

**Fig. 3:**
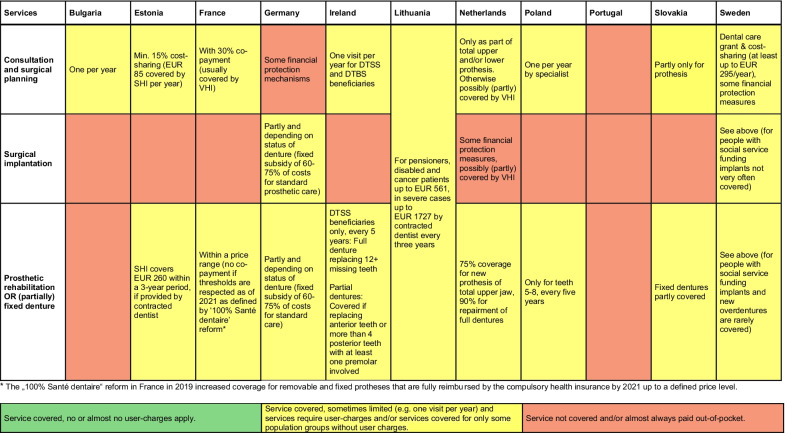
Coverage across countries for services provided in Vignette 3: Implant-borne restoration and prosthetic rehabilitation (75-year-old patient). *Notes*: empty cells summarise information using the traffic light system; *DTSS* Dental Treatment Services Scheme; *DTBS* Dental Treatment Benefit Scheme; *SHI* Social health insurance; *OOP* out-of-pocket payments; *VHI* voluntary health insurance

The information provided by individual country experts in the survey was extracted and summarised in one table per vignette (Additional file 1: Tables S1–S3), exploring each service of the patient pathway by the three dimensions (coverage, cost-sharing, and physical availability/determinants of access) per country. We further synthesised responses (Figs. 1, 2, 3 below) using a traffic light system (green–yellow–red) to visually compare results across countries. These comparative tables build the foundation for the cross-country analysis of coverage. Results on physical availability and determinants of access are broken down in more detail in Table 2 and analysed separately, as access barriers were often similar across the three vignettes.

**Table 2:**
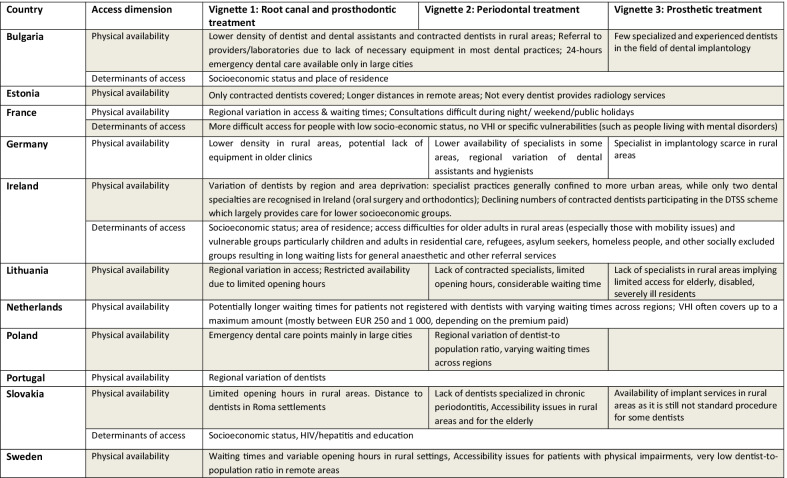
Physical availability and determinants of access

## Results

Completed vignettes were received from the 11 countries named above between October and December 2020. If answers were unclear, country experts were contacted to provide clarifications. Overall, responses varied in level of detail provided.

Some responses (in particular in Ireland and Sweden) showed the complexity of the coverage system for dental care, indicating need for further explanation. Dental services in Ireland are delivered through three publicly funded schemes: (i) the Public Dental Service (PDS), which provides emergency and some routine oral healthcare for children under the age of 16 and certain vulnerable groups, (ii) the Dental Treatment Services Scheme (DTSS) that entitles certain adults to some services free of charge, and (iii) discounted dental treatment under the Dental Treatment Benefit Scheme (DTBS) to those who have paid three years of social insurance contributions [33–35]. In addition, private dental care is available for patients that pay fully out-of-pocket and claim back fees of up to 20% of the treatment cost for certain non-routine procedures through tax relief [36].

In Sweden, dental care is free up to the age of 23 and all others receive an annual general dental care allowance between EUR 30 and EUR 60 to encourage dental check-ups and preventive care. People with certain illness or conditions (e.g. difficult-to-treat diabetes) receive a special dental care subsidy of EUR 60 every six months. In addition, most dental care in Sweden is subject to a high-cost protection scheme, which aims to protect patients from very high dental care costs. Treatment costs above certain thresholds during a twelve-month period are covered at 50% (for costs between EUR 295 and 1 470) or 85% (above EUR 1 470) of the reference prices. The Netherlands stands out in coverage of dental care by complementary voluntary health insurance (VHI). Most dental care services are not publicly covered but reimbursed in part by VHI plans, which are used by 84% of the population. In France, private insurance also plays an important role in the reimbursement of non-routine dental care services not publicly covered.

The following sections summarise results on coverage per vignette, followed by results on service access across vignettes.

### Coverage

#### Vignette 1: Urgent care with root canal and prosthodontic treatment

The first vignette explores treatment for acute pain due to caries. Related dental care services are in general covered in most responding countries, except for the Netherlands and Portugal (Fig. 1). Emergency services and radiography are covered in most countries, often with standard cost-sharing such as in France and Sweden (sometimes covered by complementary VHI) or with restrictions regarding the number of emergency visits and radiographs covered, such as in Ireland, where patients are eligible for one emergency consultation per year only. In Bulgaria, Ireland and Slovakia, emergency consultations are only covered if patients have not received another consultation that year. In the Netherlands and Portugal, emergency dental care visits as well as the other services of the vignette are not covered at all, as dental services are generally not part of the statutory benefit package.

There is a lot of variation regarding coverage of treatment alternatives of tooth extraction and root canals. Limited services and cost coverage for tooth extractions can be found in Estonia, where it is only covered in case of emergency and also in France, Lithuania and Sweden, where cost-sharing is required. In Ireland, only DTSS beneficiaries are entitled to tooth extraction. Tooth extractions are covered overall more comprehensively than root canal treatments. Root canal treatment can be excluded from coverage, such as in Bulgaria and Ireland, or be limited to certain parts of the mouth (usually covered for visible teeth, i.e. molar to molar), as in Poland. In many countries, molar root canal treatment requires substantial cost-sharing, and it can be fully excluded from public coverage for the majority of the population, as in Ireland.

Restoration with composite material and prosthodontic treatment are less comprehensively covered overall. In Germany, there is a fixed subsidy of 60% for standard treatment of crowns or onlays, which can be increased if patients are demonstrably consistent about preventive visits. The remaining costs, as well as any difference of costs due to patients choosing superior materials than those covered by insurance have to be paid out-of-pocket (OOP). In all other countries, only a fraction of the costs for fixed prosthodontic treatment is covered by the statutory health insurance. In several countries, complementary VHI seems to play an important role for the reimbursement of dental treatments that are not or only partially covered, including prosthodontic treatment.

#### Vignette 2: Chronic periodontal condition

The second vignette describes a multimorbid patient with chronic periodontitis that requires a scaling and root planning, and regular follow-up visits. Regular check-ups with the dentist seem to be less comprehensively covered across countries than the acute visit in Vignette 1. In some countries, the number of dental check-ups is capped at one per year (Bulgaria, Ireland, Slovakia, Poland) or subject to cost-sharing, such as in Estonia and France (Fig. 2). Scaling and root planning are also only partially covered in many countries or limited to a share of teeth (e.g. in Poland). Moreover, the number of planned follow-up visits to stop disease progression and stabilise bone loss are restricted in some countries (Ireland, Poland and Slovakia).

Interestingly, there are large variations in coverage of periodontal probing and elimination of dental calculus (which is part of periodontal treatment to prevent disease progression). The latter treatment is usually performed by a dental assistant or dental hygienist. In Germany, with comparatively comprehensive coverage for dental care overall, dental cleanings are not covered by the statutory health insurance, while in Slovakia (which has more limited coverage) the social health insurance covers periodontal probing and elimination of dental calculus. Basic dental hygiene in Slovakia is partly covered by SHI insurance in the case patients attend regularly preventive check-ups twice a year. In Ireland, one scale and polish per year is covered up to EUR 42 for those who contributed to social insurance in the last three years (Dental Treatment Benefit Scheme (DTBS)), corresponding to almost half of the population. Some cost-sharing applies in Estonia and Lithuania, while patients in the remaining countries (as in Germany) have to pay fully out-of-pocket for these services.

#### Vignette 3: Coverage of implant-borne restoration and prosthetic rehabilitation across countries

The third vignette describes prosthetic treatment for an older, edentulous patient who received full upper and lower dentures five years ago. Overall, the required interventions of prosthetic restoration are less comprehensively covered than services in Vignettes 1 and 2. Coverage gaps exist regarding the requirement for cost-sharing (Fig. 3). While some countries employ financial protection measures to assist lower-income individuals procure dentures (e.g. Germany, Ireland, the Netherlands), the OOP costs to be borne by patients can still be substantial. In many countries, coverage of prosthetic rehabilitation or dentures is time-bound, with coverage intervals ranging between three to five years. In Lithuania and Estonia, for example, costs for new prosthetic rehabilitation are covered up to a ceiling of EU 561 (Lithuania for pensioners, disabled and cancer patients) and EUR 260 (Estonia) every three years and if provided by contracted dentists (the exact amount covered can vary by level of bone retention). France expanded coverage of dental prostheses (including bridges, crowns and movable prosthetics) as of 2021. In Germany, surgical implantation is only covered for patients with exceptional medical indications (e.g. jaw deformities). For prosthetic rehabilitation or fixed dentures, the fixed subsidy for dentures applies that covers 60–75% of costs. Overall, implants are not covered by statutory insurance and are fully OOP in most countries.

An exception in coverage for prosthetic treatment is the Netherlands, where general dental care is usually excluded from the broad benefit package for adults. The Dutch statutory basic tariff, however, covers the cost of full dentures at a reimbursement rate of 75% for new prothesis and at 90% for the repair of full dentures, with an annual deductible of EUR 385 (this deductible also applies to other health services and has to be paid by adults before the insurer reimburses). An additional fee of EUR 250 per jaw applies, though lower jaw implants are covered under certain conditions.

### Service access: physical availability and other determinants

The results reported in the three vignettes also show that patients may experience very different kinds of physical barriers in accessing dental care (Table 2). The most important barriers reported in all three vignettes across countries relate to the availability of dental care providers, be that due to a general shortage of professionals contracting with public payers or regional variation. In Estonia, for example, the number of contracted dentists per capita is very low and represents the major limitation for access. In Ireland, the number of dentists contracted to operate in the public dental scheme is rapidly declining. Almost all countries reported a shortage of dentists, particularly in rural and remote areas as well as deprived areas with impacts for waiting times, opening hours (shorter in rural areas) and travel distances. As dentists are primarily located in urban areas, physical access to dental care for patients in rural areas is often more difficult. This compounds for interventions requiring multiple visits, making waiting times a major access barrier. In Poland, for example, the average waiting time in 2020 was 16 days, but varied from six days to 41 days across regions.

Moreover, appropriate technical equipment (e.g. X-ray units) is not equally available across dental practices, necessitating referrals to other providers or laboratories, as reported in Bulgaria. Accessibility issues for people with reduced mobility in smaller and older dental clinics were reported as another access barrier in France, Lithuania and Sweden, with an example of this being dental care facilities lacking ramps or having narrow doors and thus not accessible for wheelchair users.

While the majority of physical access barriers were similar across the three vignettes, emergency care (Vignette 1) and more specialised treatment pathways (Vignettes 2 and 3) highlight access barriers specific to specialised services and providers. Emergency dental services and out-of-office hour dental care in general are often only available in large cities in some countries (Vignette 1). The unequal distribution and/or lack of specialised dentists as well as dental hygienists constitute major barriers in many countries. In Ireland, dentists with a special interest in endodontics are generally confined to more urban areas. In Slovakia, the lack of specialists on periodontal conditions results in a low quality of care for these patients (Vignette 2). Lithuania experiences a lack of dental assistants in facilities contracted by the statutory health system. As a result, patients incur OOP costs, as the services of dental assistants are only covered if they are employed in a contracted facility. Moreover, the lack of specialists in rural areas has become a main barrier for access (Vignette 2). For Slovakia, respondents highlighted that stomatology centres are confined to larger cities, creating access barriers for patients requiring implant-based treatments and also in Bulgaria, where very few dentists are experienced in dental implantology as it is a relatively new specialty (Vignette 3).

The socioeconomic status of patients was reported as the main determinant of access to dental care in nearly all countries. This is particularly pronounced when patients have to pay upfront for services that are reimbursed retrospectively by health insurance or cover very high OOP costs. In Lithuania, for example, the high cost of dentures (Vignette 3) implies that the intervention remains unaffordable for low-income groups. Several countries have recognised that in theory, those with cognitive impairment or mental health conditions might be less able to formulate a care request or understand the different benefits and treatment processes of alternatives, such as getting a root canal vs. an extraction. In some countries, providers might deny care due to financial reasons (related to insurance status or income level).

Across all vignettes, most respondents highlighted that patient age can inhibit access and affect outcomes, for instance by needing to travel long distances. Access barriers due to difficulties with formulating the care request may be similarly exacerbated in this patient group, particularly for the third vignette, with patients potentially finding it difficult to understand the benefits of different options and/or navigate complicated administrative processes that can help with claiming support to cover OOP costs.

Other determinants may also impact access. Evidence from Sweden, for example, identified female gender, higher educational levels and native status as drivers for seeking care for chronic conditions—men, less educated people and foreigners are less likely to seek care. Foreigners and the less educated are also less likely to take advantage of cost-sharing mechanisms.

The question on the role of provider attitudes was the one most frequently left without adequate responses due to lack of relevant evidence. However, several countries reported indicative reasoning for motivating factors. Most frequently, care denial was driven by insufficient coverage (either because public coverage tariffs are too low or because patients are deemed unable to cover OOP costs) or insufficient skill on the side of the practitioner (i.e. being able to work with children, cognitively impaired patients or individuals living with a mental disorder). One country also mentioned dentists refusing care to patients with chronic infectious diseases like hepatitis C and HIV due to the associated precautions.

## Discussion

This vignette study has demonstrated the limited public coverage of several common dental services in many settings. The three vignettes exemplified the considerable variation of service and cost coverage for dental are across the 11 countries. Basic dental care, such as emergency consultations, tooth extraction and X-rays are covered in most countries without co-payments. In general, tooth extraction might be considered as the most affordable choice and therefore be more broadly covered by statutory insurance. However, this largely depends on the location of the tooth. In most cases, tooth loss creates not only deteriorating jawbone, gum disease, poor eating habits or difficulty speaking, but also reduces overall quality of life [37] and requires more expensive treatments to replace removed teeth.

Cost-sharing applies as a rule for most services in the vignettes and is structured very differently across countries. Cost-sharing may come in the form of co-insurance (such as in France), fixed subsidies (Estonia, Germany and Sweden) or as a deductible^1^ (the Netherlands, where co-payments also apply for total prothesis). The most significant cost-sharing applies to fixed prosthodontic treatment, where only a fraction of costs is covered by the statutory system and therefore these options remain unaffordable for many people. In many countries, the number of dental services covered is limited per annum (e.g. dental examination) or over a defined period of several years (for dental protheses). The specific teeth covered for some treatments can also be restricted. In most countries, statutory coverage is limited to standard materials; above-standard materials, which ensure high-quality dental care and thus better health outcomes have to be paid out-of-pocket by the patient. This showcases the general limited coverage of dental care as regards service coverage when compared to other health services. Overall, dental care seems to be subject to more cost-sharing and restrictions compared to other areas. This results in limited financial protection for the costs of oral health care in many countries (see also [13]) and financial hardship for households that also impacts the use of dental care. When comparing unmet needs for different types of care (medical care or prescribed medicines), dental care is the most frequent type that people forego due to financial reasons. On average, 14% of adults report unmet needs for dental care due to costs in EU countries [38].

Financial protection measures often address the needs of specific population groups like low-income earners or other vulnerable groups (pregnant women, children, patients with serious illness or mental or physical disabilities) [7]. Some financial protection mechanisms also exist for older people, as reported in Estonia and Lithuania, where pensioners receive higher reimbursement for prosthodontic treatments than younger adults. In Sweden, people above the age of 65 as well as individuals 24–29 years old are eligible for a general dental care grant, which is higher than for all other adults [7]. However, even mitigating measures such as the high-cost protection scheme in Sweden, do not necessarily fully alleviate OOP burdens. For services only provided in the private sector without public coverage, prices are often unregulated (e.g. Poland), and resulting OOP costs are substantial. In many countries, VHI is common for dental care (e.g. Germany, France, the Netherlands and Portugal), for (full) coverage of services or coverage of cost-sharing obligations. In the Netherlands, VHI reimbursement is capped depending on the insurance policy, incurring additional OOP costs for more expensive treatments. Older patients are particularly threatened with high(er) OOP costs, as many teeth increasingly being retained into older age are often heavily restored and/or have some degree of advanced periodontal disease [3, 25].

There is a large variation of incentives created by service coverage across countries, which can be contradictory. While dental extraction seems to be better covered than tooth retaining procedures (root canal treatment) in many countries, there are different schemes to incentivise preventive care, such as in Germany or Slovakia. In Slovakia, patients only receive a dental allowance (EUR 100 to 150 per year) towards cost-sharing requirements if they had a dental examination in the previous year. In Sweden, the general dental care grant intends to encourage adults to regularly visit their dentist for check-ups and preventative care. However, the current potential of preventive therapies in dentistry to improve oral health and contain costs is still underutilised throughout Europe. Countries need to step back from the current treatment-focused approach and create new ways of oral disease prevention and oral health promotion by strengthening the integration of oral health into primary health care [4, 5, 39, 40]. Overall, there is potential for mutual learning from existing incentive schemes that focus on preventative care as well as benefit schemes that cover dental care more comprehensively.

In all countries, statutory coverage of dental care does not necessarily imply that people have unrestricted access to dental care services. Many similar barriers limit access to dental care across countries, which relates to the physical availability of care (due to long distance, poor quality, reduced opening hours, waiting times) as well as a person’s ability to obtain necessary care or the attitude of the provider. In particular, the limited availability of contracted dentists creates a major access barrier to public dental care in many countries. This is especially detrimental for patients residing in rural areas or less wealthy regions that may not profit from the same density of professionals, specialised clinics or modern equipment as those residing in urban centres. The impact of geographical imbalances of dental care providers highlights the need for a more diversified skill mix among oral health care professionals and improved workforce planning.

Another interesting element is the lack of consideration of physical accessibility for people with disabilities in older, more remote facilities (e.g. wheelchair access) and the potential difficulties of patients with cognitive impairment or other types of dependency to understand the benefits and disadvantages of different care options, adhere to treatment plans or navigate the complicated reimbursement system. New policies to improve oral health should take these factors into account in workforce education and capacity planning.

Barriers to high-quality care in some countries are also attributable to the lagging establishment of “best practices”. In Vignette 3, newer prosthetic treatments involving surgical implants were not widely reported as available in all countries. In Slovakia, for example, implants are still not a standard procedure for some dentists and thus the physical availability of the service is worse in some parts of the country. A lack of a respective dental guideline may be the major reason for these non-harmonised treatment pathways. At the European level, there is currently no detailed, common guidance concerning management and treatment of patients with oral health problems, complicating the comparison of coverage and access to oral health services.

This vignette study on coverage and access to dental care has several strengths and limitations. On the one hand, it demonstrated the potential of the vignette approach to pick up access barriers usually not demonstrated by performance assessment indicators and exemplified the variations and complexities of dental care coverage. It confirmed previous knowledge about the limited coverage of dental services, which automatically pre-disposes patients from lower socioeconomic strata to experiencing further barriers along the path to realised access, widening health inequalities. The study also showed the impact of a limited or unbalanced supply of dental care providers on access to care, even among eligible individuals and for covered services. At the same time, the study has several limitations. A clear limitation of vignettes is that they may not accurately reflect the real world, both with regard to the textual descriptions of used case examples and the elicited hypothetical behaviour [21, 41]. The comprehensiveness and accuracy of information relied on the knowledge and experience of respondents. Participating experts may not always have comprehensive knowledge on each dental procedure covered in the vignettes, the relevant regulations of coverage, or the effective access to these services. There was also substantial variation in the detail level of responses. Moreover, due to lack of harmonised dental guidelines, the treatment pathways described in the vignettes did not necessarily correspond to the usual treatment options in some countries. Thus, it became clear that responses could have been skewed by the initial focus of the vignette template on coverage, as categories further to right of the table related to realised access were not always tackled in detail. This was probably also compounded by the background of respondents (see methods section). For this exercise on dental care, it is conceivable that the three chosen vignettes were too many in terms of services included to be answered at once, as a certain level of respondent fatigue was obvious for the third vignette on edentulism (less granularity, more skipped fields in the template).

Based on the results of our work, future studies should investigate the association of (limited) coverage and access with the burden of oral diseases more closely. This might be hampered by the limited availability of comparable data on oral health measures within and across European countries, which in itself constitutes a call for additional funding for data collection. The role of different incentive models for preventative oral health services and the extent to which evidence on (cost-)effectiveness guides decisions on dental benefit baskets should also be further explored to guide the formulation of future policies.

## Conclusion

The results of the vignettes reveal that statutory coverage of dental care varies across 11 European countries, but access barriers are largely similar. Statutory coverage of many dental services is limited, and substantial cost-sharing applies in most countries, leading to high OOP spending. Socioeconomic status is thus a main determinant for access to dental care, though other factors such as geography, age and comorbidities can inhibit access and affect outcomes. Additionally, different incentive structures have implications on how patients are treated regarding state-of-the-art dental care.

Furthermore, our findings showed that coverage in most oral health systems is targeted at treatment and less at preventative oral health care. Policies are needed that exploit the potential of preventive oral care and favour its integration into existing strategies for the prevention and control of NCDs, which have major risk factors and social determinants in common. Enhanced integration of oral health care with medical care is also needed to better meet the needs of the growing population of older adults with multiple health conditions.

The study showed that the vignette approach revealed important gaps in access that would have stayed under the radar when only looking at available services in the benefit basket and thus remains interesting for further research. Finally, our approach revealed the lack of common guidelines in the field of dentistry at national and European levels. Developing common guidelines and promoting best practice rules that dentists in the EU adhere to are important. A major prerequisite for this is an evidence base for dental guidelines that is established and internationally agreed upon.

## Supplementary Information

**Additional file 1.** Supplementary tables 1–4.

## Data Availability

Not applicable.

## Abbreviations

DTSS: Dental treatment services scheme
DTBS: Dental treatment benefit scheme
EU: European Union
NCD: Non-communicable disease
OOP: Out-of-pocket
PDS: Public dental service
SHI: Social health insurance
YLD: Years lived with disability
VHI: Voluntary health insurance

## Footnotes

1 In the Netherlands, an annual deductible of incurred health care costs (EUR 385) has to be paid by the user before the insurer begins to reimburse for health and dental services.
