# Supplementary material for: Exploring variation of coverage and access to dental care for adults in 11 European countries: a vignette approach
Source: BMC Oral Health. 2022 Mar 9;22:65. doi: 10.1186/s12903-022-02095-4 (PMC8905841; doi:10.1186/s12903-022-02095-4)
Supplement: Supplementary file 1 — Additional file 1. Supplementary tables 1–4. [file 12903_2022_2095_MOESM1_ESM.docx]

**Supplementary tables 1-4**

| **Vignette 1** | | ***Services in patient pathway*** | | | | | |
| --- | --- | --- | --- | --- | --- | --- | --- |
| **Country** | **Dimension** | **Emergency consultation with dentist** | **Radiography (bitewing)  X-rays** | **Root canal treatment OR** | **Tooth extraction** | **(Interim) Reconstruction with composite material** | **Fixed prosthodontic treatment (crown/onlay)** |
| **Bulgaria** | *Coverage* | x (one per year) | - | - | x | Obturation with amalgam or chemical composite | - |
|  | *Cost-sharing* | Fixed co-pay | OOP | OOP | - | Fixed co-pay (EUR 2/BGN 4),  OOP for composite material | OOP |
|  | *Physical availability/ Determinants of access* | - Lower density of dentist and dental assistants and contracted dentists in less densely populated areas, which results in differences in access due to distance - Referral to providers/laboratories due to lack of necessary equipment of most dental practices; 24-hours emergency dental care available only in large cities - Socioeconomic status and place of residence main determinants of access | | | | | |
| **Estonia** | *Coverage* | X (only contracted dentists) | X (only contracted dentists) | x (partially) | x (in case of emergency) | x (partially) | x (partially) |
|  | *Cost-sharing* | - | 50%  (EUR 40 covered per year | 50%  (EUR 40 covered per year) | 50%  (EUR 40 covered per year) | 50%  (EUR 40 covered per year) | 50%  (EUR 40 covered per year) |
|  | *Physical availability/ Determinants of access* | Only contracted dentists covered; Longer distances in remote areas; Not every dentist provides radiology services | | | | | |
| **France** | *Coverage* | x (70%) | x (70%) | x (70%) | x (70%) | x (70%) | x (70% with an upper limit) |
|  | *Cost-sharing* | 30% (usually covered by VHI), some exemptions | 30% (usually covered by VHI), some exemptions | 30% (usually covered by VHI), some exemptions | 30% (usually covered by VHI), some exemptions | 30% (usually covered by VHI), some exemptions | VHI cover costs partly |
|  | *Physical availability/ Determinants of access* | Regional variation in access & waiting times; More difficult access for people with low socio-economic status, no VHI or specific vulnerabilities (such as people living with mental disorders) ; Consultations difficult during night/ weekend/public holidays | | | | | |
| **Germany** | *Coverage* | x | x | x | x | x (only certain materials) | x (basic treatment) |
|  | *Cost-sharing* | - | - | (high quality treatment) | - | Top-up for better material | 60% of basic treatment, top-up for better material; higher coverage for low-income |
|  | *Physical availability/ Determinants of access* | Lower density in rural areas | Potential lack of equipment in older clinics | Regional differences of OOP requirement | - | - | Delays on reimbursement approval depending on SHI |
| **Ireland** | *Coverage* | One visit/ year (for DTSS and DTBS beneficiaries) | One visit/ year (for DTSS and DTBS beneficiaries ) | - | - (except for DTSS scheme beneficiaries) | - (except amalgam covered for DTSS scheme beneficiaries) | - |
|  | *Cost-sharing* | OOP (no OOP for 1 covered visit; means-testing for lower income groups) | OOP (no OOP for 1 covered visit) | OOP | OOP | OOP | OOP |
|  | *Physical availability/ Determinants of access* | - Number of DTSS contracted dentists declining nationwide resulting in difficulties in access for lower socioeconomic groups)Access difficulties for older adults in rural areas especially with mobility issues - Significant difficulties in accessing oral healthcare for those who are most vulnerable or who require complex care i.e. those in residential facilities, refugees, asylum seekers, homeless people and other socially excluded groups - Specialist practice confined to urban areas | | | | | |
| **Lithuania** | *Coverage* | X (only contracted dentists) | X (only contracted dentists) | X (only contracted dentists) | X (only contracted dentists) | X (only contracted dentists) | X (only contracted dentists) |
|  | *Cost-sharing* | OOP (for fillings and disposables, except vulnerable groups) | OOP (for fillings and disposables, except vulnerable groups) | OOP (for fillings and disposables, except vulnerable groups) | OOP (for fillings and disposables, except vulnerable groups) | OOP (for fillings and disposables, except vulnerable groups) | OOP (for fillings and disposables, except vulnerable groups) |
|  | *Physical availability/ Determinants of access* | Regional variation in access; Restricted availability due to limited opening hours; physical accessibility for severely disabled | | | | | |
| **Netherlands** | *Coverage* | - | - | - | - | - | - |
|  | *Cost-sharing* | VHI/OOP (some financial protection measures) | | | | | |
|  | *Physical availability/ Determinants of access* | Potentially longer waiting times for patients not registered with dentists | | | | | |
| **Poland** | *Coverage* | x | 2 radiographs/per year | For front teeth | x | For front teeth | - |
|  | *Cost-sharing* | - | - | Depends on location | - | Depends on location | OOP |
|  | *Physical availability/ Determinants of access* | Regional variation: emergency dental care points mainly in large cities; more generous benefits for certain vulnerable groups | | | | | |
| **Portugal** | *Coverage* | - | - | - | -  (few services available at public clinics) | - | - |
|  | *Cost-sharing* | VHI/OPP | VHI/OOP | VHI/OOP | VHI/OOP | VHI/OOP | VHI/OOP |
|  | *Physical availability/ Determinants of access* | Regional variation of dentists | | | | | |
| **Slovakia** | *Coverage* | -  (except contracted dentists) | x (partly) | x (partly) | x | x (partly) | x (partly) |
|  | *Cost-sharing* | OOP (for non-contracted dentists; OOP can also be asked by contracted dentists) | OOP (for non-contracted dentists; OOP can also be asked by contracted dentists) | OOP (for non-contracted dentists; OOP can also be asked by contracted dentists) | - | OOP (for non-contracted dentists; OOP can also be asked by contracted dentists) | OOP (for non-contracted dentists; OOP can also be asked by contracted dentists) |
|  | *Physical availability/ Determinants of access* | - Problems in rural areas and Roma settlements - HIV/hepatitis and education main determinants of access, socioeconomic status | | | | | |
| **Sweden** | *Coverage* | x (partly) | x (partly) | x (partly) | x (partly) | x (partly) | x (partly) |
|  | *Cost-sharing** | Variable (dental grant, high-cost protection scheme and financial protection scheme apply) | Variable (dental grant, high-cost protection scheme and financial protection scheme apply) | Variable (dental grant, high-cost protection scheme and financial protection scheme apply) | Variable (dental grant, high-cost protection scheme and financial protection scheme apply) | Variable (dental grant, high-cost protection scheme and financial protection scheme apply) | variable (dental grant, high-cost protection scheme and financial protection scheme apply), for social service funding limited to crowns and onlays on third molar |
|  | *Physical availability/ Determinants of access* | - Waiting times and variable working hours in rural settings - Accessibility issues for patients with physical impairments - Very low dentist densities in remote areas | | | | | |

Notes: BGN – Bulgarian Lev; DTSS - Dental Treatment Services Scheme (31% of the population are entitled to this scheme, to qualify one’s weekly income must fall below a certain threshold); DTBS - Dental Treatment Benefit Scheme (49% of the population, all people who have paid three years of social insurance contributions); OOP – out-of-pocket payment; VHI – Voluntary health insurance
* A dental care grant of either 300 SEK or 600 SEK per year (depending on age) is provided to all adults as of age of 24 to cover dental check-ups and preventative care. Treatment costs beyond 3 000 SEK are partially covered by high-cost protection scheme. The financial protection scheme applies to people with very low income, persons with certain chronic disabilities (special needs) are getting the treatment at a very low cost or for free.

| **Vignette 2** | | ***Services in patient pathway*** | | | |
| --- | --- | --- | --- | --- | --- |
| **Country** | **Dimension** | **Scheduled visit with the dentist** | **Curettage (performed by a dentist)** | **Periodontal probing and elimination of dental calculus**  **OR** | **Regular follow-up visits** |
| **Bulgaria** | *Coverage* | x (one per year) | - | - | - |
|  | *Cost-sharing* | - | OOP | OOP | OOP |
|  | *Physical availability/ Determinants of access* | - Lower density of dentist and dental assistants and contracted dentists in less densely populated areas, which results in differences in access due to distance - Referral to providers/laboratories due to lack of necessary equipment of most dental practices; 24-hours emergency dental care available only in large cities - Socioeconomic status and place of residence main determinants of access | | | |
| **Estonia** | *Coverage* | x (partially) | x (partially) | x (partially) | x (partially) |
|  | *Cost-sharing* | Minimum of 15%, EUR 85 per year paid by EHIF | Minimum of 15%, EUR 85 per year paid by EHIF | Minimum of 15%, EUR 85 per year paid by EHIF | Minimum of 15%, EUR 85 per year paid by EHIF |
|  | *Physical availability/ Determinants of access* | - Only contracted dentists covered - Longer distances in remote areas | | | |
| **France** | *Coverage* | x (70%) | - | - | x (70%) |
|  | *Cost-sharing* | 30% (usually covered by VHI), some exemptions | OOP (VHI covers partly costs, large price variation) | OOP/VHI | 30% (usually covered by VHI), some exemptions |
|  | *Physical availability/ Determinants of access* | - Regional variation in access and waiting times - More difficult access for people with low socio-economic status, no VHI or specific vulnerabilities (such as people living with mental disorders) Consultations difficult during night/weekend/public holidays | | | |
| **Germany** | *Coverage* | x | x (partially, two required dental cleanings mostly not covered) | x | x |
|  | *Cost-sharing* | - | Two dental cleanings | OOP for dental cleaning | - |
|  | *Physical availability/ Determinants of access* | Lower density in rural areas, difficult for patients in nursing homes | Availability of specialist required for complex procedure varies by location | Regional variation of dental assistants and hygienists | - |
| **Ireland** | *Coverage* | x (one visit per year for DTSS and DTBS beneficiaries) | DTSS: x (4 visits for exceptional/high risk cases); DTBS: x (scale and polish: EUR 42) | DTSS: x-(exceptional/high risk cases)  DTBS: x (UR 42) | DTSS: x (4 visits/ year) DTBS: x (one visit/year) |
|  | *Cost-sharing* | OOP (no OOP for 1 covered visit) | DTSS: - ; DTBS: OOP | DTSS: - ; DTBS: OOP (except for EUR 42 | DTSS: OOP (no OOP for 4 visits/year for exceptional/high risk cases)  DTBS: OOP (no OOP for 1 visit/ear) |
|  | *Physical availability/ Determinants of access* | - Access to low income scheme reducing as Number of DTSS contracted dentist declines - Access difficulties for the most vulnerable i.e. older people with mobility issues especially rural areas, those in residential facilities or socially excluded groups including homeless people - Periodontics is not a recognized dental specialty in Ireland | | | |
| **Lithuania** | *Coverage* | x (only contracted dentists) | X (only contracted dentists) | X (only contracted dentists) | X (only contracted dentists) |
|  | *Cost-sharing* | OOP (for fillings and disposables, except vulnerable groups) | OOP (if not under referral) | OOP (for disposables, except vulnerable groups, and if dental assistant is not employed by contracted facility) | OOP (for disposables, except vulnerable groups)- |
|  | *Physical availability/ Determinants of access* | - Regional variation in accessibility of dentists/ dental assistants - Lack of physical accessibility for severely disabled patients - Lack of contracted specialists - Referral for the specialist - Limited opening hours - Financial hardship | | | |
| **Netherlands** | *Coverage* | - | - | - | - |
|  | *Cost-sharing* | VHI/OOP (some financial protection measures) | VHI/OOP (some financial protection measures) | VHI/OOP (some financial protection measures) | VHI/OOP (some financial protection measures) |
|  | *Physical availability/ Determinants of access* | - Threatening shortage of dentists in rural areas | | | |
| **Poland** | *Coverage* | x (provided once a year calendar) | x (within 25% of teeth) | - | x (three times/ calendar year) |
|  | *Cost-sharing* | - | Anaesthesia and dressing | OOP | - |
|  | *Physical availability/ Determinants of access* | - Regional variation of dentist-to population ratio, varying waiting times across regions | | | |
| **Portugal** | *Coverage* | -   VHI/OOP (few services available in public primary clinics) | -   VHI/OOP (few services available in public primary clinics ) | -   VHI/OOP (few services available in public primary clinics) | -   VHI/OOP (few services available in public primary clinics) |
|  | *Cost-sharing* | VHI/OOP | VHI/OOP (for pensioners with low income partially covered) | VHI/OOP | VHI/OOP |
|  | *Physical availability/ Determinants of access* | - Regional variation of dentists’ density (lowly populated regions have low densities) | | | |
| **Slovakia** | *Coverage* | x (partly) | x (partly) | x | x (two times a year) |
|  | *Cost-sharing* | Co-payment (except for yearly check-up) | Co-payment | - | OOP (except for two covered follow-ups a year) |
|  | *Physical availability/ Determinants of access* | - Lack of dentists specialized in chronic periodontitis (paradentologists) - Accessibility issues in rural areas and for the elderly, with lack of transport links, Limited opening hours - Socioeconomic status, HIV/hepatitis and education main determinants of access | | | |
| **Sweden** | *Coverage* | x | x | x | x |
|  | *Cost-sharing** | Variable (dental grant, high-cost protection scheme and financial protection scheme apply) | Variable (dental grant, high-cost protection scheme and financial protection scheme apply) | Variable (dental grant, high-cost protection scheme and financial protection scheme apply) | Variable (dental grant, high-cost protection scheme and financial protection scheme apply) |
|  | *Physical availability/ Determinants of access* | - Accessibility issues for patients with physical impairments - Very low dentists densities in remote areas - Waiting times and variable working hours in rural settings | | | |

Notes: BGN – Bulgarian Lev; DTSS - Dental Treatment Services Scheme (31% of the population are entitled to this scheme, to qualify one’s weekly income must fall below a certain threshold); DTBS - Dental Treatment Benefit Scheme (49% of the population, all people who have paid three years of social insurance contributions); OOP – out-of-pocket payment; VHI – Voluntary health insurance
* A dental care grant of either 300 SEK or 600 SEK per year (depending on age) is provided to all adults as of age of 24 to cover dental check-ups and preventative care. Treatment costs beyond 3 000 SEK are partially covered by high-cost protection scheme. The financial protection scheme applies to people with very low income, persons with certain chronic disabilities (special needs) are getting the treatment at a very low cost or for free.

| **Vignette 3** | | ***Services in patient pathway*** | | |
| --- | --- | --- | --- | --- |
| **Country** | **Dimension** | **Consultation and surgical planning** | **Surgical implantation** | **Prosthetic rehabilitation OR (partially) fixed dentures** |
| **Bulgaria** | *Coverage* | x (one per year) | - | - |
|  | *Cost-sharing* | Co-payment (EUR 0.9/BGN 1.80) | OOP | OOP |
|  | *Physical availability/Determinants of access* | - Very few specialized and experienced dentists in the field of dental implantology; Socioeconomic status and place of residence | | |
| **Estonia** | *Coverage* | x (partially) | - | x (partially) |
|  | *Cost-sharing* | Minimum of 15%, EUR 85 per year paid by EHIF | OOP | Amounts exceeding 260 EUR in a 3-year period |
|  | *Physical availability/ Determinants of access* | Only contracted dentists covered, and longer distances in remote areas | Longer distances in remote areas | Only contracted dentists covered, and longer distances in remote areas |
| **France** | *Coverage* | x (70%) | - | x (up to 100% within a price range) |
|  | *Cost-sharing* | 30% (usually covered by VHI), some exemptions | OOP/VHI | VHI for those over the price threshold, different generosity depending on contract |
|  | *Physical availability/ Determinants of access* | - Regional variation in access and waiting times - More difficult access for people with low socio-economic status, no VHI or specific vulnerabilities (such as people living with mental disorders) Consultations difficult during night/weekend/public holidays | | |
| **Germany** | *Coverage* | - | X (partly, depending on status of denture) | X (partly, if considered as standard care and depending on status of denture) |
|  | *Cost-sharing* | OOP | fixed subsidy of 60-75% of costs for standard care (top-up OOP for better material or full OOP), financial protection mechanisms | fixed subsidy of 60-75% of costs for standard care if medically necessary, financial protection mechanisms |
|  | *Physical availability/ Determinants of access* | Specialist in implantology scarce in rural areas, x-ray devices needed for good surgical planning not always available | - | - |
| **Ireland** | *Coverage* | x (one visit per year for DTSS and DTBS beneficiaries) | - | DTSS: basic complete dentures every 5 years DTBS: - |
|  | *Cost-sharing* | OOP (no OOP for 1 covered visit, but if specialist is oral surgeon/prosthodontist, generally full price applies) | OOP | DTSS: OOP except dentures every 5 years DTBS: OOP |
|  | *Physical availability/ Determinants of access* | - Number of DTSS contracted dentists declining nationwide resulting in difficulties in access for lower socioeconomic groups - Access difficulties for older adults in rural areas especially with mobility issues - Significant difficulties in accessing oral healthcare for those who are most vulnerable or who require complex care i.e. those in residential facilities, refugees, asylum seekers, homeless people and other socially excluded groups   Most specialists located in urban areas | | |
| **Lithuania** | *Coverage* | x (for pensioners, disabled, cancer patients; if consulted by primary care dentist) | x (partially for pensioners, disabled, cancer patients: in contracted dental practices) | x (partially for pensioners, disabled, cancer patients, every three years in contracted dental practices) |
|  | *Cost-sharing* | - | Co-payment for pensioners (difference between costs and maximum of EUR 504.81(and EUR 1 031 if teeth 4,5, 6 in lower jaw is affected) | Co-payment for pensioners (difference between costs and maximum of EUR 504.81(and EUR 1 031 if teeth 4,5, 6 in lower jaw is affected) |
|  | *Physical availability/ Determinants of access* | - Regional variation in accessibility of contracted dentists/specialists - Lack of physical accessibility for severely disabled patients | | |
| **Netherlands** | *Coverage* | - (unless part of total upper and/or lower prosthesis) | - | x (75% for new prosthesis of total upper jaw, 90% for repairment of full dentures) |
|  | *Cost-sharing* | OOP (unless part of total upper and/or lower prosthesis) | - | OOP (25% for new prothesis of total upper jaw, 10% for repair, 100% for partial prosthesis and implants, with mandatory deductible of EUR 385**) |
|  | *Physical availability/ Determinants of access* | Long waiting times depending on workload of specialists | Long waiting times depending on workload of specialists | Long waiting times depending on workload of specialists |
| **Poland** | *Coverage* | X (one examination by specialist) | - | x (only partial denture (for teeth 5-8), once in 5 years) |
|  | *Cost-sharing* | OOP (except for one covered examination) | OOP (unregulated prices) | - |
|  | *Physical availability/ Determinants of access* | - | - | - |
| **Portugal** | *Coverage* | - | - | -   (few services available in public primary clinic) |
|  | *Cost-sharing* | VHI/OOP (few services available in public primary clinic) | VHI/OOP, no exceptions for pensioners with low income | VHI/OOP |
|  | *Physical availability/ Determinants of access* | Regional variation of dentists | - | - |
| **Slovakia** | *Coverage* | x (partly, only in regard to prosthesis not implants) | - | x (fixed dentures partly covered, implants not covered) |
|  | *Cost-sharing* | OOP (implants assessment) | OOP | OOP (implants and variable on fixed dentures) |
|  | *Physical availability/ Determinants of access* | - Availability of implant services in rural areas as it is still not standard procedure for some dentists - Accessibility issues in rural areas - Socioeconomic status and HIV/hepatitis main determinants of access | | |
| **Sweden** | *Coverage* | x | x | x |
|  | *Cost-sharing** | Variable ((dental grant, high-cost protection scheme and financial protection scheme apply depending on socioeconomic conditions) | Variable (dental grant, high-cost protection scheme and financial protection scheme apply depending on socioeconomic conditions)) | Variable (dental grant, high-cost protection scheme and financial protection scheme apply depending on socioeconomic conditions) |
|  | *Physical availability/ Determinants of access* | - Accessibility issues for patients with physical impairments - Very low dentists’ densities in remote areas - Waiting times and variable working hours in rural settings | | |

Notes: BGN – Bulgarian Lev; DTSS - Dental Treatment Services Scheme (31% of the population are entitled to this scheme, to qualify one’s weekly income must fall below a certain threshold); DTBS - Dental Treatment Benefit Scheme (49% of the population, all people who have paid three years of social insurance contributions); OOP – out-of-pocket payment; VHI – Voluntary health insurance
* A dental care grant of either 300 SEK or 600 SEK per year (depending on age) is provided to all adults as of age of 24 to cover dental check-ups and preventative care. Treatment costs beyond 3 000 SEK are partially covered by high-cost protection scheme. The financial protection scheme applies to people with very low income, persons with certain chronic disabilities (special needs) are getting the treatment at a very low cost or for free.

** The mandatory deductible of EUR 385 is a fixed amount of incurred health care costs to be paid by the user before the insurer begins to reimburse for services (it does not apply to GP care, maternity care, district nursing, and care for

children under the age of 18).

**Table 4.**

| **Dental care vignettes (dental caries, periodontic conditions, edentulism)** |  | **Coverage** | | **Access** | | | **Determinants of access** |
| --- | --- | --- | --- | --- | --- | --- | --- |
|  | **Services** | **Is the service covered by the statutory system?** (including exemptions) | **Does cost-sharing** (value or rule for determining the amount) apply? Any **financial protection** measures (e.g. lower cost-sharing for low-income groups/chronic patients, annual cost-sharing caps etc.)? | **Is there a lack of *physical availability* of services** (e.g. due to distance, lack of statutory/contracted providers, poor quality of services, limited opening hours, waiting times and waiting lists) | Do patients lack the ***ability to obtain* necessary care?*** (e.g. incapacity to formulate care request) | Do patients face problems due to the **attitude of the provider?**** (discrimination, care denial, inability to accommodate preferences) | **Can you think of any factors that would worsen/improve access of this particular vignette? (**e.g. age, sex, and socioeconomic status, insurance status, legal status, place of residence, night/day or ANYTHING else |
| **Vignette 1** A 35-year-old patient has not been able to sleep for two nights due to a strong, beating pain in the right-lower jaw. He requests an urgent appointment with his r dentist. The dentist determines that the patient needs a root-canal treatment to preserve the first lower molar and treat the pain. The patient decides for the root canal treatment and against the alternative of tooth extraction. Following the root canal treatment, reconstruction with composite material is used until a fixed prosthodontic treatment (crown/onlay) can be placed. | *Emergency consultation with dentist* |  |  |  |  |  |  |
|  | *Radiography ((bitewing) X-rays)* |  |  |  |  |  |  |
|  | *Root canal treatment*  ***OR*** *Tooth extraction* |  |  |  |  |  |  |
|  | *(interim) reconstruction with composite material* |  |  |  |  | - |  |
|  | *Fixed prosthodontic treatment (crown/onlay)* |  |  |  |  |  |  |
| **Vignette 2**  A 66 year old patient with co-morbidities (obesity, diabetes) has frequent discomfort in the upper jaw. After a consultation, chronic periodontitis with generalized level 2 mobility is diagnosed, requiring surgical curettage, one dental extraction and frequent follow-ups to stop disease progression and stabilize bone-loss. | *Scheduled visit with the dentist* |  |  |  |  |  |  |
|  | *Curettage (performed by a dentist)* |  |  |  |  |  |  |
|  | *Periodontal probing, and elimination of dental calculus (performed by dental assistant)* |  |  |  |  |  |  |
|  | *Regular follow-up visits* |  |  |  |  |  |  |
| **Vignette 3**  An edentulous 75-year-old patient received upper and lower full-dentures 5 years ago. She feels she has lost significant capacity to chew as the inferior prosthesis is poorly retained and gets displaced when speaking or eating. She seeks counseling from her dentist, who recommends two implants on the lower anterior jaw and an overdenture to increase retention. She agrees with this course of treatment and against more sophisticated (partially) fixed alternatives. | *Consultation and surgical planning* |  |  |  |  |  |  |
|  | *Surgical implantation* |  |  |  |  |  |  |
|  | *Prosthetic rehabilitation: New prosthesis or adjustment of old prosthesis using the implants*  ***OR***  *(partially) fixed dentures* |  |  |  |  |  | - |

******* due to a person’s incapacity to formulate care request, obtain the care or to apply for coverage (and fulfil the necessary requirements) due to their condition or situation (e.g. people with cognitive impairment, mentally ill, homeless); ** for example due to discrimination (on age, gender, race, religious beliefs, sexual orientation, etc) leading to care denial or inability to accommodate care to the patient’s preferences.
